# Revisiting the Glucose Kinase Superfamily: From Origins to the Evolution of Metabolism

**DOI:** 10.1093/gbe/evaf221

**Published:** 2025-11-28

**Authors:** Douglas Jardim-Messeder, Lúcia Barzilai, Gilberto Sachetto-Martins

**Affiliations:** Programa de Biologia Molecular e Biotecnologia, Instituto de Bioquímica Médica Leopoldo de Meis, Universidade Federal do Rio de Janeiro—UFRJ, Rio de Janeiro, Brazil; Departamento de Genética, Instituto de Biologia, Universidade Federal do Rio de Janeiro—UFRJ, Rio de Janeiro, Brazil; Departamento de Genética, Instituto de Biologia, Universidade Federal do Rio de Janeiro—UFRJ, Rio de Janeiro, Brazil; Departamento de Genética, Instituto de Biologia, Universidade Federal do Rio de Janeiro—UFRJ, Rio de Janeiro, Brazil

**Keywords:** glucokinase, hexokinase, ribokinase, ROK, glucolysis

## Abstract

The glucose kinase superfamily, which includes enzymes such as hexokinases and glucokinases, plays a central role in energy metabolism across all domains of life. This study explores their evolutionary origins, functional diversity, and adaptation to ecological niches, tracing their journey from early life forms to modern organisms. Recent advances reveal how these enzymes have diversified, with some retaining broad specificity, while others evolved high substrate specificity, reflecting the metabolic demands of their environments. By integrating phylogenetic, structural, and functional analyses, this work sheds light on the evolutionary pressures that shaped these enzymes and their role in metabolic innovation, not only deepening our understanding of life's biochemical evolution but also connecting ancient metabolic pathways to contemporary cellular processes.

Significance While glucose phosphorylation is a central step in cellular metabolism, the evolutionary origin and diversification of the enzymes that catalyze this process remain poorly understood. This study reveals how distinct glucose kinases—such as hexokinase and glucokinase—evolved in different domains of life, and how key endosymbiosis events shaped their distribution and function in modern eukaryotes. These findings help explain how metabolic pathways have adapted to diverse environments over evolutionary time, providing new insights into enzyme specialization and metabolic flexibility.

## Organization of the Glucose Kinases Superfamily

To maintain their high degree of organization, the living organisms must acquire and transform energy through the metabolism (Schrödinger 1944; Lehninger 1970). During the evolution, metabolic pathways did not arise randomly but were shaped by natural selection during the evolution of species (Darwin 1859). The importance of metabolic pathways lies not only in their role as sources of essential elements but also in their function of extracting energy from high-energy molecules.

**Fig. 1.**
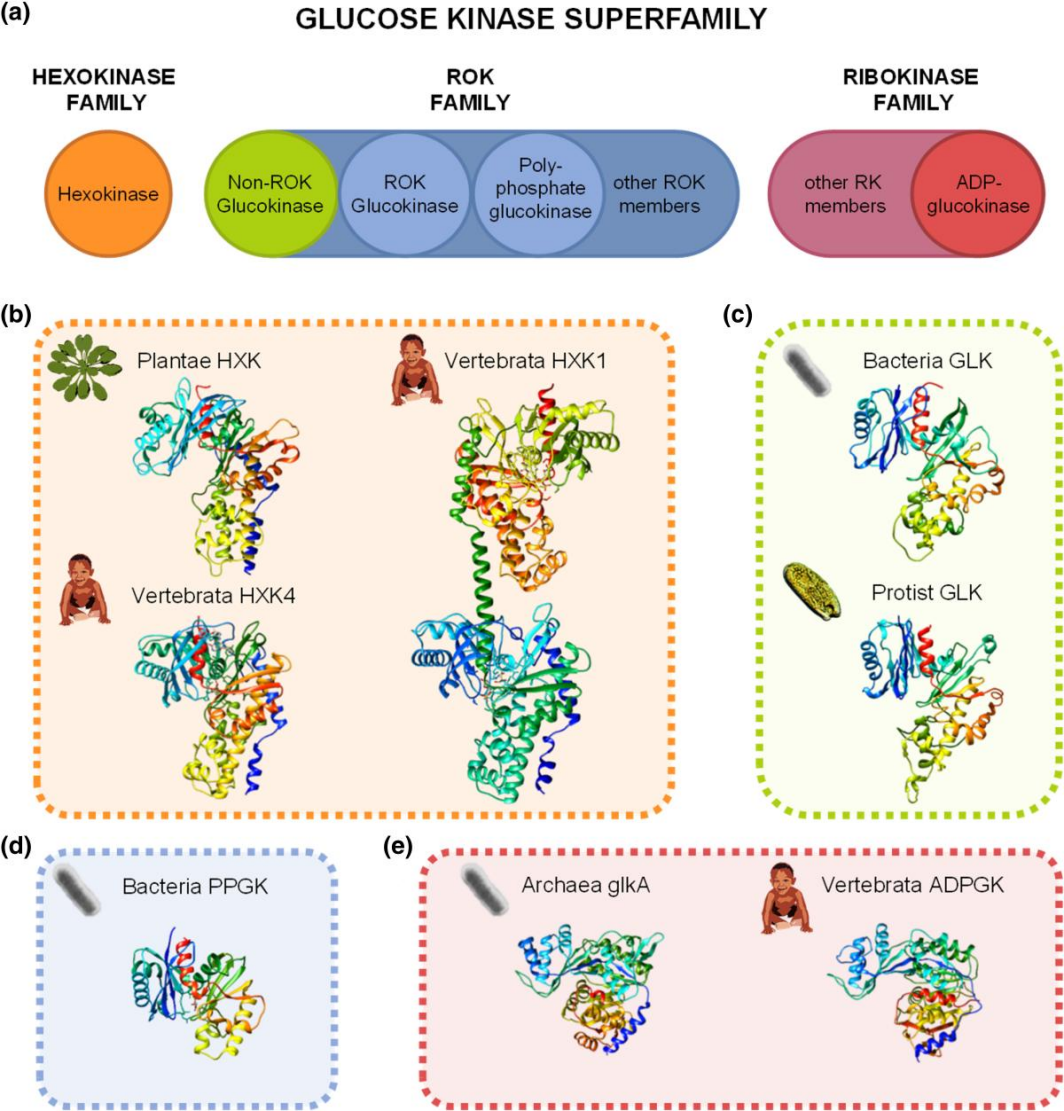
Glucose kinase superfamily. a) Proposed classification of the Glucose Kinase superfamily. The glucose kinase superfamily can be divided into the hexokinase, ROK, and ribokinase families. The hexokinase family includes all hexokinases found in eukaryotes. The ROK family comprises non-ROK glucokinases, ROK glucokinases, PPGMK, and other ROK proteins, such as specific sugar kinases and transcriptional repressors. The ribokinase family includes ADP-glucokinases from Archaea and animals, as well as other related proteins. b) Structural representation of different hexokinases from eukaryotes: plant Arabidopsis HXK1 (NP_194642.1), vertebrate human HXK4 (NP_000153.1), and vertebrate human HXK1 (NP_001309294.1). c) Structural representation of non-ROK glucokinases from prokaryotes and eukaryotes: prokaryote *Escherichia coli* GLK (NP_416889.1) and eukaryote *Paramecium tetraurelia* GLK (XP_001456377.1). d) Structural representation of Actinomycetota *Mycobacterium* sp. PPGMK (PJE19729.1). e) *Pyrococcus horikoshii* ADPGK (O58328.1) human ADPGK (NP_112574.3). The prediction of three-dimensional models was performed by Alphafold Protein Structure Database using default parameters.

The phosphorylation of glucose is the first step of its utilization in most cells. Four different enzymes catalyze this reaction: hexokinase (EC 2.7.1.1), glucokinase (EC 2.7.1.2), polyphosphate gluco(manno)kinase (PPGMK, EC 2.7.1.63), and ADP-dependent glucokinase (EC 2.7.1.147). These enzymes exhibit varying efficiency and specificity, being distributed across different organisms, and belong to distinct families (Fig. 1).

In most eukaryotes, glucose is phosphorylated by hexokinases, which can also phosphorylate other hexoses, such as fructose, mannose, and galactose (Cárdenas et al. 1998). In fungi (Maitra 1970), invertebrates (Ureta et al. 1987), and plants (Jang et al. 1997), hexokinase isoforms typically have a molecular mass of 50 kDa. Vertebrate tissues express up to five isoenzymes (Katzen et al. 1965; Irwin and Tan 2008), which the evolution involved duplication and fusion of an ancestral 50 kDa hexokinase, leading to 100 kDa enzymes with duplicated domains, such as hexokinase I, hexokinase II, hexokinase III, and the hexokinase domain-containing protein (HKDC) (Cárdenas et al. 1998). The hexokinase IV, however, retains the molecular mass of 50 kDa, originated from a posterior fission and deletion of one of the domains (Ureta 1982). Despite being commonly referred to as “glucokinase,” hexokinase IV is not specific for glucose, making this designation misleading (Cárdenas et al. 1998).

The “true” glucokinase is found only in bacteria and a few eukaryotes, such as green algae (Chlorophyta), red algae (Rhodophyta), and protist algae (Ureta et al. 1987; Middleton 1990). The glucokinases are members of the ROK (Repressor, ORF, Kinase) family, and they are classified into Group A and Group B. Glucokinases from Group A are found in Gram-negative bacteria, cyanobacteria, and eukaryotic algae and are also referred to as non-ROK glucokinases due to the loss of the characteristic ROK domain. Group B includes glucokinases with ROK domain and found in Archaea and Gram-positive bacteria (Wu et al. 2001; Kawai et al. 2005). Despite this, the active site residues involved in glucose binding have remained fully conserved in both ROK and non-ROK glucokinases (Conejo et al. 2010).

The Group B also comprises sugar kinases specific to other carbohydrates, such as *N*-acetylglucosamine kinase (NagK; EC 2.7.1.59), *N*-acetylmannosamine kinase (NanK; EC 2.7.1.60), fructokinases (FK, MAK; EC:2.7.1.4), and allokinase (AlsK; EC 2.7.1.55); and transcription repressors, such as *N*-acetylglucosamine utilization regulator (NagC) and MAKING LARGE COLONIES protein (Mlc), previously described as regulatory members of Group B within the ROK family (Titgemeyer et al. 1994; Kawai et al. 2005; Bréchemier-Baey et al. 2015).

The PPGMK enzymes are also found in Group B and use polyphosphates as phosphate donors to phosphorylate glucose and, to a lesser extent, mannose (Kawai et al. 2005). Unlike glucokinases, which are widely distributed across prokaryotes and some eukaryotes, PPGMK is found exclusively in specific bacterial groups, including Actinobacteria, Acidobacteria, Bacteroidetes, and Verrucomicrobia (Albi and Serrano 2015; Rivas et al. 2018).

The presence of nonspecific hexokinases in eukaryotes, while bacteria possess highly specific sugar kinases, may seem to contradict the idea that enzyme specificity has increased over evolutionary time—where highly specialized enzymes are believed to have evolved from more generalist ancestral proteins. However, simpler organisms do not necessarily have fewer specific enzymes. Regardless of their complexity, all modern organisms have been shaped by natural selection to thrive in specific ecological niches, with enzyme specificity reflecting their functional requirements. Despite their brief lifespan, bacterial populations experience multiple generations with a relatively constant supply of sugars. These conditions can collectively generate a greater selective pressure for enzymatic specificity in bacteria compared to eukaryotes (Cárdenas et al. 1998).

In some Archaea, there is also an unusual glucokinase (Ureta et al. 1987) called glkA. Like glucokinases, it is highly specific for glucose but uses ADP, instead of ATP, as a phosphate donor (Ureta et al. 1987; Kengen et al. 1995). The usage of ADP is proposed to be related to the ability of Archaea to activate sugars under starvation conditions when energy levels are low (Cárdenas et al. 1998). The ADP-dependent glucokinase is also present in animals and is thought to have been acquired in an ancestral organism through lateral gene transfer from an Archaeal species (Ronimus and Morgan 2004), providing advantages under nutrient-deprived and anoxic conditions (Richter et al. 2012). ADP-dependent glucokinase belongs to the Ribokinase family and is not evolutionarily related to hexokinase or glucokinase (Rivas et al. 2018).

## Phylogenetic Relationships of Glucose Kinases Superfamily

In eukaryotes, hexokinase appears to be originated from a ∼50-kDa dimeric ATPase (Bork et al. 1992; Cárdenas et al. 1998). Although bacterial glucokinases have been tentatively suggested as potential ancestors, the evolutionary relationship between glucokinase and hexokinase remains unknown. Despite sequence comparisons showing no significant similarity, structural comparisons and the similar size of the hypothetical 25-kDa ancestor suggest a possible shared evolutionary history (Finn et al. 2006; Rivas et al. 2018).

To better understand the evolutionary history of glucokinase and other members of the ROK family and the emergence of glucokinase in eukaryotes, a phylogenetic analysis was performed with 137 protein sequences representing different members of ROK family, including GLK, NagK, NanK, FK, AlsK, NagC, and Mlc. The ROK sequences were retrieved through BLASTP analysis in the NCBI database using as bait the sequences from *Escherichia coli* (Conejo et al. 2010). The ID number of identified sequences is indicated in Table S1. The sequences were aligned in MEGA 11: Molecular Evolutionary Genetics Analysis version 11 (Tamura et al. 2021) using MUSCLE tool. The final dataset was used for phylogenetic tree reconstruction using the maximum likelihood method implemented in IQ-TREE, under the best-fit substitution model. Node support was assessed with 1,000 ultrafast bootstrap replicates and the approximate likelihood-ratio test, providing accurate and reproducible trees under well-fitted models with a reasonable computational cost.

**Fig. 2.**
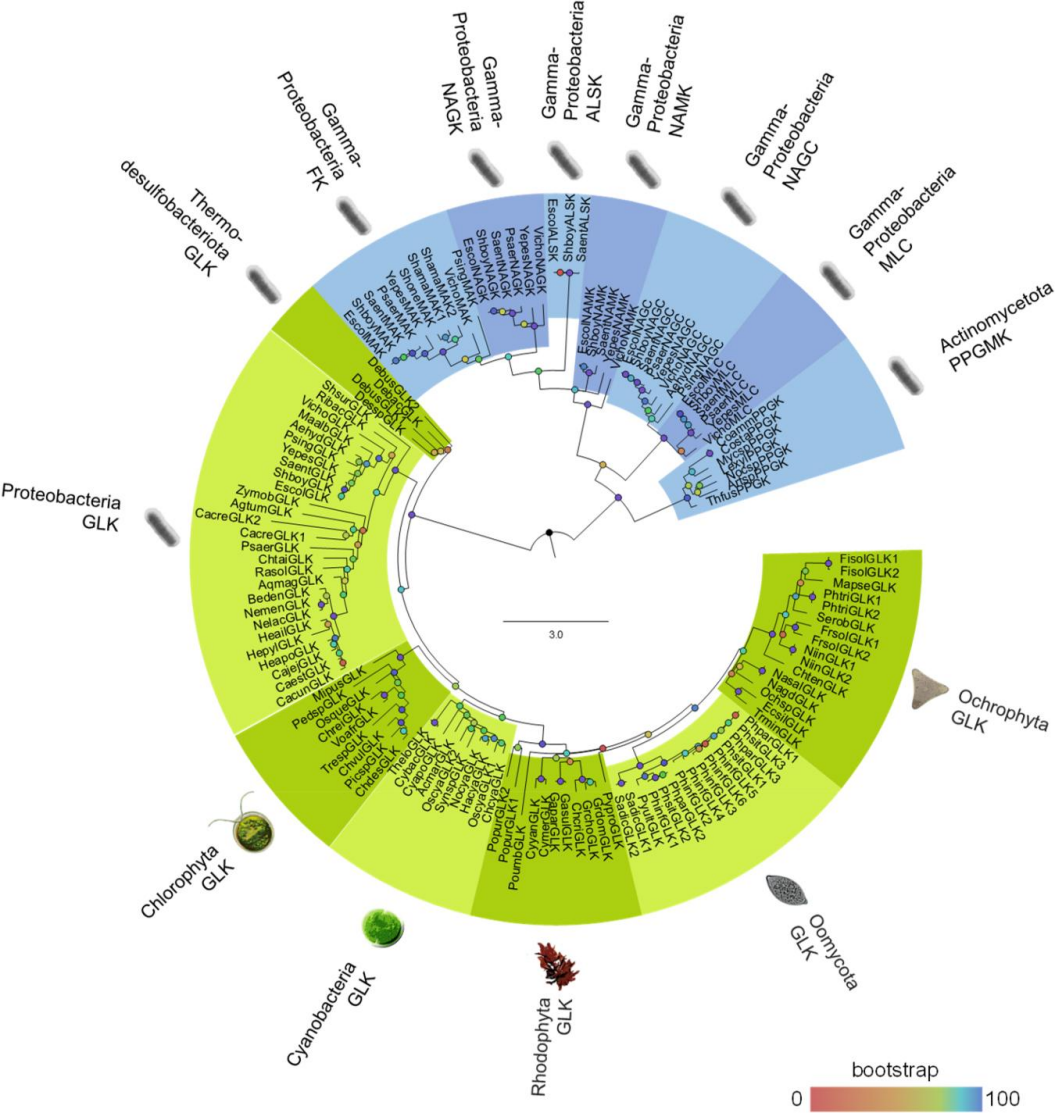
Phylogenetic analysis of sugar kinases and transcriptional repressors from family ROK. For the phylogenetic analysis, we selected 137 protein sequences representing different enzymes and transcriptional repressors of ROK family and manually inspected them for ambiguous positions, which were subsequently removed. A total of 347 amino acid positions were included in the final dataset for phylogenetic tree reconstruction using maximum likelihood method under the best model selection in IQ-TREE2 with 1000 replicates of bootstrap statistics. The values of bootstrap are indicated by a color scale. The non-ROK GLKs (Group A) from Thermodesulfobacteriota, Proteobacteria, Cyanobacteria, Chlorophyta, Rhodophyta, and the Chromalveolate lineages Oomycota and Ochrophyta, as well as other sugar kinases (NagK, NanK, FK, and AlsK), the transcription repressors NagC and Mlc from Proteobacteria, and PPGK from Actinomycetota are indicated in the figure.

The phylogenetic topology confirms the division of the ROK family into Group A and Group B (Kawai et al. 2005) (Fig. 2). This split represents the most ancestral dichotomy within the family, indicating that glucokinase, which belongs to Group A, likely diverged independently of the other sugar kinases and transcriptional repressors. Additionally, glucokinases are distributed across different eukaryotic lineages. Subsequent gene duplication events and neofunctionalizations within Group B appear to have given rise to kinases with specificity for different sugars, while the later acquisition of a DNA-binding domain at the N-terminus led to the evolution of carbohydrate-responsive transcriptional repressors. According to Conejo et al. (2020), specific substitutions in the active site residues determine carbohydrate recognition and substrate discrimination within the ROK protein family. This is a significant evolutionary event, as it expanded the range of carbon sources and enabled more precise control of gene expression in response to prokaryote organisms to metabolic needs. The tree shows that NagC and Mlc form a monophyletic group, suggesting they diverged from a common ancestor after the acquisition of the DNA-binding motif.

The ROK family likely originated early during evolution, and it remains unclear whether an enzyme resembling this ancestor still exists or in which organism. Additionally, many ROK family members remain poorly annotated in most microorganisms, impairing a more comprehensive phylogenetic analysis. Previous works have proposed that the origins of the ROK family may be linked to PPGMK (Mukai et al. 2004; Kawai et al. 2005), but phylogenetic analysis does not support this idea (Fig. 2).

The origin of the ROK family remains uncertain and may never be fully resolved. However, models of the origin of life provide us with some insights. Although the fermentation of carbohydrates has long been considered among the earliest catabolic pathways (Oparin 1924; Krebs 1981; Lazcano 2016), it has been suggested that the “primordial soup” was relatively rich in amino acids (Orgel 1973), whereas carbohydrates were significantly less abundant (Broda 1975; Larralde et al. 1995). Despite evidence for the prebiotic synthesis of small sugar molecules (Ritson and Sutherland 2012), as well as nonenzymatic carbohydrate formation via the formose reaction (Rauchfuss 2008), the production and accumulation of significant amounts of carbohydrates likely remained highly challenging until the establishment of autotrophic organisms. Consequently, glycolysis as we know it today arose relatively late in Earth history (Rivas et al. 2018). In such an environment, selective pressure likely favored enzymes with relatively high sugar affinity but broad substrate specificity. Over time, metabolic activity likely altered the sugar composition, creating new selective pressures.

The Archaea glucokinase was demonstrated to be able to phosphorylate different hexoses at nearly the same rate and with similar affinity to glucose (Shakir et al. 2023). Thus, it is possible that this enzyme retains characteristics of the potential ancestor of the ROK family, which probably showed low specificity compared to modern bacterial enzymes and even eukaryotic hexokinases. Furthermore, one could imagine ancestral prebiotic “cells” operating with a limited set of enzymes, each possessing low specificity, enabling them to interact with a wide array of related substrates.

## Origin and Evolution of Sugar Kinases in Eukaryotes

The phylogenetic analysis shows that glucokinases from Cyanobacteria form a monophyletic group with glucokinase from eukaryotes, such as Chlorophyta, Rhodophyta, and protist algae (Fig. 2). This topology strongly suggests a common origin and that glucokinases were possibly acquired by eukaryotes through the endosymbiosis event that gave rise to the chloroplast from a cyanobacterial ancestor. This process enabled photosynthesis and introduced genes of cyanobacterial origin into the host lineage. Primary chloroplast endosymbiosis led to the diversification of three major lineages of Archaeplastida: Viridiplantae (including Chlorophyta and Embryophyta), Rhodophyta, and Glaucophyta.

On the other hand, the presence of hexokinase in both Bikont and Unikont groups, which are the main eukaryotic lineages, suggests that it emerged early in eukaryotic evolution and was possibly present in the eukaryotic host. Based on the distribution of hexokinase and glucokinase genes across the major eukaryotic lineages, it is expected that the Archaeplastida ancestor initially possessed both the eukaryotic hexokinase and the bacterial glucokinase. However, during evolution, Chlorophyta retained both hexokinase and glucokinase, while Rhodophyta retained only glucokinase, and only hexokinase is found in land plants. Due to the lack of well-annotated genomes and functional studies, it is not yet possible to determine the enzymes present in the Glaucophyta lineage.

Different cyanobacterial and eukaryotic algae strains exhibit remarkable metabolic flexibility, allowing them to adapt their growth strategies and switch between photoautotrophic, mixotrophic, or heterotrophic modes, depending on the presence of an exogenous carbon source (Shihira and Krauss 1965; Johnson and Alric 2012; Lucius and Hagemann 2024).

In chlorophyte, hexokinase has been identified as a master regulator of photosynthesis and carbon metabolism in response to exogenous glucose, inducing degradation of photosynthetic apparatus, and reduction of thylakoid membranes (Roth et al. 2019a, 2019b). These data suggest that hexokinase from chlorophytes exerts a pivotal role as a glucose sensor, as demonstrated in yeast and plants (Rolland et al. 2006; Sheen 2014). Rhodophyte algae can also modulate their trophic state in response to carbon source availability (Gross and Schnarrenberger 1995), suggesting the presence of sugar sensors distinct from hexokinase.

The evolutionary pressures that led to the retention of hexokinase and glucokinase in chlorophytes, while hexokinase was lost in rhodophytes, remain unclear. One possible explanation could be the different ecological niches and metabolic strategies employed by these algae. Chlorophytes, which are primarily freshwater or marine organisms, may rely on hexokinase for efficient glucose sensing and utilization in environments with more varied carbon sources. In contrast, rhodophytes, which often inhabit more extreme environments, where the presence of other carbon-sensing pathways or metabolic strategies, such as those involving glucokinase, might be more beneficial.

Differences in starch accumulation mechanisms could also provide important insights into the evolution of sugar kinases in eukaryote algae. Chlorophyta, like land plants, synthesize and store starch in the chloroplast stroma, in a mechanism similar to glycogen synthesis in bacteria, reflecting evolutionary origin from endosymbiotic cyanobacteria (Bhattacharya and Medlin 1995; Reith 1995). In contrast, rhodophytes synthesize and store a distinct polysaccharide, known as “floridean starch,” in cytosolic granules (Meeuse et al. 1960), a process analogous to glycogen synthesis in fungi and animals (Pueschel 1990; Viola et al. 2001). This divergence in starch synthesis and compartmentalization in Chlorophyta and Rhodophyta likely resulted from selective pressures on the machinery inherited from either the endosymbiont or the original host, respectively.

These differences lead to distinct subcellular sugar availability in these lineages. In chlorophytes, sugars can originate from starch degradation within the chloroplast or from direct uptake from the environment into the cytosol. In contrast, both sources of sugars in rhodophytes are confined to the cytosol. Consequently, differences in sugar kinase activity can be expected between these groups.

Experimental data on the subcellular localization of sugar kinases in different algae lineages are not available in the literature. However, chlorophyte glucokinases are predicted to chloroplast stroma, whereas hexokinase is predicted to be a cytosolic enzyme. Hence, the endosymbiosis process enabled chlorophytes to acquire sugar kinases localized in these distinct subcellular compartments. In the chloroplast, the glucose-specific glucokinase acts by phosphorylating the glucose derived from starch degradation, while the cytosolic hexokinase acts on a broader variety of sugars derived from the environment.

In rhodophytes, glucokinase is predicted to be a cytosolic enzyme, able to phosphorylate glucose derived from floridean starch degradation. The absence of starch metabolism in the chloroplast may contribute to the cytosolic localization of glucokinase, which is smaller and structurally simpler than hexokinase, making its maintenance energetically more favorable.

Chromalveolata algae also exhibit bacterial glucokinase and lack homologs to hexokinase. The chromalveolates are a polyphyletic eukaryotic supergroup that includes several photosynthetic lineages (Keeling 2009). These protists evolved through secondary endosymbiosis with an ancestral rhodophyte and possess chloroplasts with multiple membranes (Moustafa et al. 2009; Dorrell et al. 2017).

The phylogenetic analysis reveals that glucokinases from Chromalveolata, including those in the Oomycota and Ochrophyta classes, form a monophyletic group with rhodophyte sequences (Fig. 2). This relationship is consistent with the evolutionary history of these lineages, which share a red-alga-derived plastid acquired through secondary endosymbiosis. Glucokinase homologs have been identified only in these plastid-bearing groups, supporting a common origin from the ancestral red algal endosymbiont. Although the evolutionary pressures driving the retention of glucokinase or hexokinase in these organisms remain unclear, we can speculate that limited access to sugars other than glucose favored the retention of glucokinase.

Previous studies have shown that red algae contributed numerous genes of endosymbiotic origin to chromalveolates, including plastid-targeted proteins involved in translation, plastid division, and biogenesis, as well as enzymes required for chlorophyll biosynthesis and energy metabolism (Li et al. 2005). In addition, ancient red algae such as *Porphyridium purpureum* acted as key mediators of gene transfer, introducing hundreds of prokaryotic and early Plantae genes into chromalveolates and other lineages through secondary and tertiary endosymbiosis (Qiu et al. 2013).

The evolution of Apicomplexa is another interesting case that highlights the relationship between the evolution of sugar kinases and the metabolic adaptations of organisms. Apicomplexa possess a highly reduced plastid organelle known as the apicoplast, which is considered a remnant plastid acquired through a tertiary endosymbiosis event involving a Chromalveolata Ochrophyta ancestor (Fussy and Oborník 2017). However, in this case, photosynthesis was not established, and Apicomplexa evolved to a group of obligate host-associated protists, primarily composed of intracellular parasites with access to a variety of sugar sources. In this lineage, the retention of the ancestral eukaryotic hexokinase and the loss of the endosymbiont-derived glucokinase likely reflect both the evolutionary simplification of the apicoplast and the adaptation to a parasitic lifestyle.

Land plants also retained hexokinase rather than glucokinase. In these organisms, successive duplications and neofunctionalization events led to the expansion of various gene families, including hexokinase, generating isoforms targeted to different cellular locations and capable of phosphorylating different hexoses in the cytosol and chloroplast. In this context, the selective pressure to retain glucokinase was diminished.

**Fig. 3.**
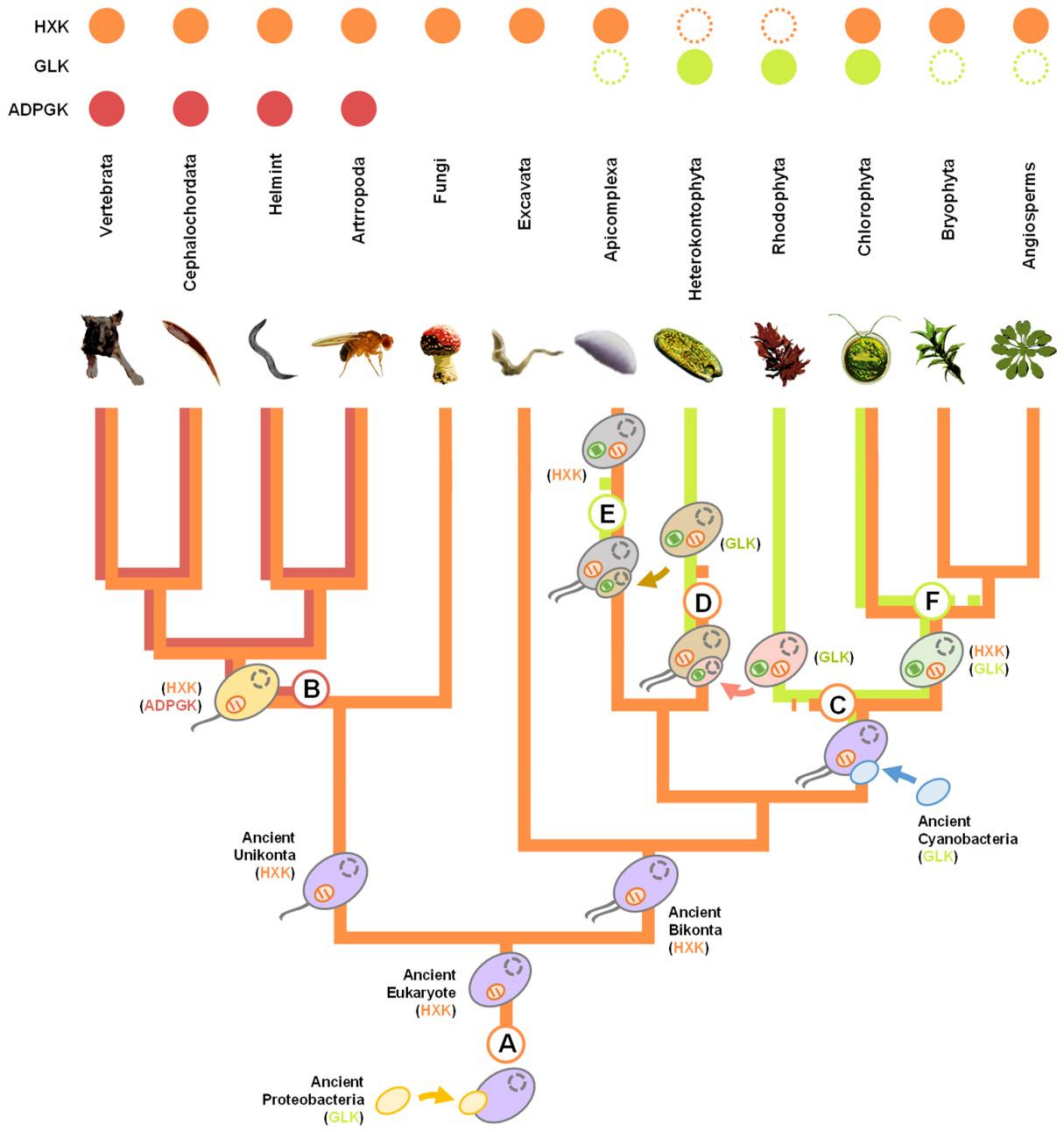
Schematic representation of the eukaryotic life tree. a) Early in eukaryotic evolution, a primary endosymbiosis event enabled the acquisition of mitochondria from an engulfed proteobacterium, which likely possessed a GLK as a glucose kinase. Ancestral eukaryotes developed the first HXK before the divergence between the Unikonta and Bikonta lineages. b) In the Unikonta branch, the ancestral animal likely acquired the ADPGK gene from an Archaeal organism through a horizontal gene transfer event. Nevertheless, the possibility that ADPGK was already present in the Archaeal host that gave rise to eukaryotes cannot be excluded. In this scenario, the gene would have been inherited from the earliest eukaryotic ancestor and subsequently lost independently in all other eukaryotic lineages, being retained only in animals. Consequently, modern animals possess both HXK and ADPGK enzymes. c) In the Bikonta branch, another primary endosymbiosis event led to the emergence of chloroplasts from engulfed cyanobacteria, which likely possessed GLK. This process enabled photosynthesis, introduced cyanobacterial-origin genes into the Archaeplastida host lineage, and gave rise to Viridiplantae (including Chlorophyta and land plants) and Rhodophyta. In the Rhodophyta lineage, the HXK gene was lost, whereas GLK was retained. d) In the Heterokontophyta lineage, a secondary endosymbiosis event led to the emergence of chloroplasts from an engulfed Rhodophyta ancestor, bringing with it a GLK gene, which was retained, while HXK was lost. e) In the Apicomplexa lineage, a tertiary endosymbiosis event led to the emergence of the apicoplast, a remnant plastid acquired from an engulfed Ochrophyta ancestor, which likely brought a GLK gene. However, in this case, GLK was lost, and HXK was retained as a glucose kinase. f) In the Viridiplantae lineage, both GLK and HXK were maintained in Chlorophyta, whereas only HXK was preserved in land plants, such as Bryophyta and Angiosperms.

Plant hexokinases are encoded by a multigene family and can be targeted to the chloroplast stroma, soluble in cytosol, or embedded in the mitochondrial outer membrane (Nilsson et al. 2011). The attachment of hexokinase to the mitochondria provides a significant evolutionary advantage by granting the enzyme direct access to mitochondrial ATP produced through oxidative phosphorylation. Vertebrates also developed mitochondrial membrane-bound hexokinase isoforms, illustrating a case of convergent evolution driven by distinct mechanisms. In plants, hexokinase is anchored to the mitochondrial membrane via a transmembrane peptide, whereas in vertebrates, it associates with the mitochondrial outer membrane primarily through its interaction with the voltage-dependent anion channel (Pedersen et al. 2002). The evolution of glucose kinases in eukaryotic life tree is indicated in Fig. 3.

## Concluding Remarks

The glucose kinase superfamily exemplifies the intricate interplay between enzyme evolution, metabolic adaptation, and ecological specialization. From the earliest stages of life to the complex metabolic networks of modern organisms, these enzymes have been pivotal in shaping cellular energy dynamics and carbon utilization. Despite significant progress in understanding their roles, several questions remain unresolved in this area, presenting important opportunities for future research that could lead to relevant project themes across various targets.

The coexistence of broad-specificity enzymes in some lineages and highly specialized kinases in others highlights the adaptive flexibility of metabolic systems. This duality underscores the role of ecological and evolutionary pressures in shaping enzyme function and specificity, enabling organisms to respond to fluctuating nutrient availability and environmental challenges. The evolutionary trajectories of glucose kinases not only offer clues about the origins of life but also contribute to fields such as metabolic engineering, biotechnology, and therapeutic development.

## Supplementary Material

evaf221_Supplementary_Data

## Data Availability

The data underlying this article are available in the article and in its online Supplementary material.
